# Systematic evaluation of oligodeoxynucleotide binding and hybridization to modified multi-walled carbon nanotubes

**DOI:** 10.1186/s12951-017-0288-z

**Published:** 2017-07-17

**Authors:** Anika Kaufmann, Silke Hampel, Christiane Rieger, David Kunhardt, Darja Schendel, Susanne Füssel, Bernd Schwenzer, Kati Erdmann

**Affiliations:** 1 Chair of Biochemistry, Department of Chemistry, Technische Universität Dresden, Bergstraße 66, 01069 Dresden, Germany; 2 Leibniz Institute for Solid State and Materials Research Dresden, Helmholtzstraße 20, 01069 Dresden, Germany; 3 Department of Urology, University Hospital Carl Gustav Carus, Technische Universität Dresden, Fetscherstraße 74, 01307 Dresden, Germany; 4 Department of Nanostructured Materials, Leibniz Institute for Polymer Research, Hohe Straße 6, 01069 Dresden, Germany

**Keywords:** Antisense oligodeoxynucleotides, Biocompatibility, Bladder cancer, Carbon nanotubes, Carrier strand, Functionalization, Hybridization, Mucoadhesion, Urothelium

## Abstract

**Background:**

In addition to conventional chemotherapeutics, nucleic acid-based therapeutics like antisense oligodeoxynucleotides (AS-ODN) represent a novel approach for the treatment of bladder cancer (BCa). An efficient delivery of AS-ODN to the urothelium and then into cancer cells might be achieved by the local application of multi-walled carbon nanotubes (MWCNT). In the present study, pristine MWCNT and MWCNT functionalized with hydrophilic moieties were synthesized and then investigated regarding their physicochemical characteristics, dispersibility, biocompatibility, cellular uptake and mucoadhesive properties. Finally, their binding capacity for AS-ODN via hybridization to carrier strand oligodeoxynucleotides (CS-ODN), which were either non-covalently adsorbed or covalently bound to the different MWCNT types, was evaluated.

**Results:**

Pristine MWCNT were successfully functionalized with hydrophilic moieties (MWCNT-OH, -COOH, -NH_2_, -SH), which led to an improved dispersibility and an enhanced dispersion stability. A viability assay revealed that MWCNT-OH, MWCNT-NH_2_ and MWCNT-SH were most biocompatible. All MWCNT were internalized by BCa cells, whereupon the highest uptake was observed for MWCNT-OH with 40% of the cells showing an engulfment. Furthermore, all types of MWCNT could adhere to the urothelium of explanted mouse bladders, but the amount of the covered urothelial area was with 2–7% rather low. As indicated by fluorescence measurements, it was possible to attach CS-ODN by adsorption and covalent binding to functionalized MWCNT. Adsorption of CS-ODN to pristine MWCNT, MWCNT-COOH and MWCNT-NH_2_ as well as covalent coupling to MWCNT-NH_2_ and MWCNT-SH resulted in the best binding capacity and stability. Subsequently, therapeutic AS-ODN could be hybridized to and reversibly released from the CS-ODN coupled via both strategies to the functionalized MWCNT. The release of AS-ODN at experimental conditions (80 °C, buffer) was most effective from CS-ODN adsorbed to MWCNT-OH and MWCNT-NH_2_ as well as from CS-ODN covalently attached to MWCNT-COOH, MWCNT-NH_2_ and MWCNT-SH. Furthermore, we could exemplarily demonstrate that AS-ODN could be released following hybridization to CS-ODN adsorbed to MWCNT-OH at physiological settings (37 °C, urine).

**Conclusions:**

In conclusion, functionalized MWCNT might be used as nanotransporters in antisense therapy for the local treatment of BCa.

**Electronic supplementary material:**

The online version of this article (doi:10.1186/s12951-017-0288-z) contains supplementary material, which is available to authorized users.

## Background

Bladder cancer (BCa) is the most common malignancy of the urogenital system and the ninth most common cancer worldwide and thus poses a great challenge to the health care sector [1, 2]. BCa treatment depends on tumor stage and prognosis of the patient. Superficial, non-muscle-invasive BCa is initially treated by transurethral resection of the bladder tumor followed by a local chemotherapy (mitomycin C, epirubicin, doxorubicin) and/or immunotherapy (*Bacillus Calmette*–*Guérin*) in order to lower the probability of recurrence and progression [1]. This adjuvant therapy is directly instilled through a catheter into the bladder, which is ideally suitable for such an intravesical drug delivery due to its hollow structure [3]. Compared to a systemic drug administration, intravesical therapy ensures higher drug concentrations at the tumor site while minimizing systemic side effects [3].

However, the intravesical drug application has to overcome some obstacles. The most prominent drawback is the low dwell time of the therapeutics due to continuous urine production and flushing during voiding. Furthermore, poor adhesion of the drugs to the urothelium and deprived penetration into it result in a decreased absorption and lower effective concentration of the therapeutic. Consequently, repeated instillations via catheterizations are required, which is inconvenient for the patient and may cause inflammatory reactions, bladder irritation and infections [3]. Finally, failure of chemotherapy could be evoked by the up-regulation of genes associated with drug resistance, which in turn provide attractive targets for molecular therapy [4, 5]. Complementary to conventional treatment options such as chemotherapy, surgery and radiation, therapeutic nucleic acids including antisense oligodeoxynucleotides (AS-ODN) and small interfering RNAs (siRNAs) have emerged as a promising strategy to modify the expression of such tumor-related genes. In order to achieve an effective therapy, it is important to sufficiently deliver the genetic cargo to the tumor cells while evading clearance and degradation. Consequently, a wide variety of viral and non-viral gene therapy vectors have been developed and evaluated in the last years.

The development of innovative drug delivery systems combining a multifunctional therapy approach is necessary and of utmost interest. Particularly, carbon nanotubes (CNT) possess immense potential as nanocarriers for such biomedical applications due to their ability to be functionalized with various therapeutic agents [6–8]. For that matter, we have shown that multi-walled CNT (MWCNT) could be loaded with conventional chemotherapeutics and thus mediate anti-proliferative effects in various cancer cells [9, 10]. The functionalization of CNT with AS-ODN or siRNAs represents another promising approach to create multifunctional nanocarriers and to sufficiently deliver genetic payload to the tumor site [6, 8]. We and others have previously shown that CNT can successfully be coupled with AS-ODN or ODN [11–14]. In our prior study, AS-ODN against the angiogenic growth factor VEGF were linked via adsorbed carrier strand oligodeoxynucleotides (CS-ODN) onto hydroxyl group-functionalized MWCNT (MWCNT-OH) [11].

In BCa it is necessary to transport the therapeutic agent to the cancer cells located in the bladder urothelium. Therefore, the gene therapy vector has to adhere to the bladder urothelium and release its genetic payload. Furthermore, it would be advantageous, if the aforementioned low dwell time of therapeutics inside the bladder could be increased. In addition to adhesion to the urothelium, cellular uptake and biocompatibility are important prerequisites for the effectiveness of nanocarriers in BCa treatment. We have previously demonstrated that various types of pristine MWCNT (p-MWCNT) can adhere to the urothelium of mouse bladders [15] and can be internalized by cancer cells [11, 16]. Another important challenge that has to be addressed when using CNT for biomedical applications in vivo is their toxic, inflammatory and immunogenic potential. Due to their small size and structural resemblance to asbestos, CNT could evoke deleterious effects, whereupon MWCNT are generally less toxic than single-walled CNT (SWCNT) [7]. However, by the introduction of functional moieties to p-MWCNT their biocompatibility as well as dispersibility, cellular uptake and mucoadhesive properties might be further improved.

In the present study we modified p-MWCNT with different functional groups (MWCNT-OH, -COOH, -NH_2_, -SH) followed by a comprehensive physicochemical characterization of the functionalized MWCNT including an evaluation of their dispersibility. Furthermore, the biocompatibility, cellular uptake and mucoadhesive properties of all MWCNT were examined. Then, we investigated their binding capacity for CS-ODN by using two different attachment strategies: non-covalent adsorption and covalent binding. Subsequently, the hybridization efficiency and the release of AS-ODN coupled to MWCNT-bound CS-ODN were evaluated at experimental and physiological conditions.

## Methods

### Chemicals

HNO_3_, NaCl and NaOH were obtained from VWR International GmbH (Darmstadt, Germany). 1-Ethyl-3-(3-dimethylaminopropyl)carbodiimid (EDC), *N*-hydroxysuccinimid (NHS), 2-(*N*-morpholino)ethanesulfonic acid (MES), tetrahydrofuran (THF) and cysteamine were purchased from Sigma-Aldrich (Taufkirchen, Germany). 3,4-dihydroxybenzaldehyde (DHBA), *N*,*N*-dimethylformamide (DMF), H_2_SO_4_, ethylenediamine, methanol and chloroform were acquired from Merck (Darmstadt, Germany). *N*-(tri(hydroxymethyl)methyl)glycin (tricine) was obtained from Alfa Aesar (Karlsruhe, Germany). Disuccinimidyl suberate (DSS) and *N,N′*-dicyclohexylcarbodiimide (DCC) were from Thermo Fisher Scientific (Dreieich, Germany) and Interchim (Montluçon, France), respectively. The buffer ingredients KH_2_PO_4_, K_2_HPO_4_·3 H_2_O, Na_2_HPO_4_·2 H_2_O, NaHCO_3_ and C_2_H_3_NaO_2_·3 H_2_O were purchased from Gruessing (Filsum, Germany). All buffers were prepared in distilled water and sterilized before use.

### Synthesis and characterization of functionalized MWCNT

**Fig. 1:**
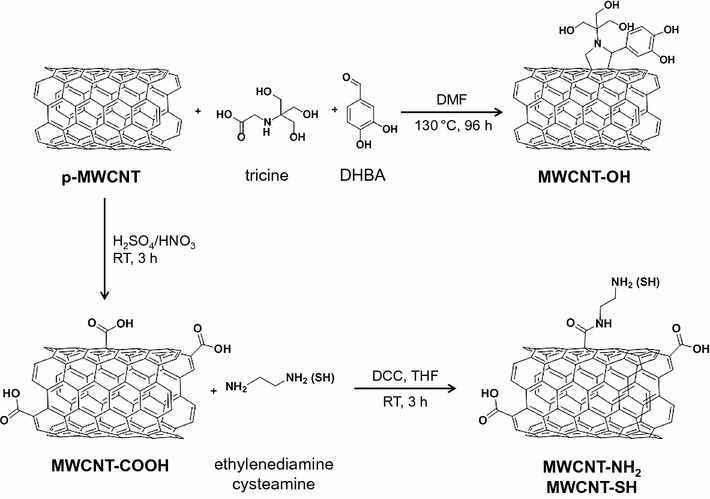
Scheme for the preparation of functionalized MWCNT from pristine MWCNT. DCC: *N,N′*-dicyclohexylcarbodiimide; DHBA: 3,4-dihydroxybenzaldehyde; DMF: *N*,*N*-dimethylformamide; RT: room temperature; THF: tetrahydrofuran

p-MWCNT were synthesized by chemical vapor deposition as described previously [15, 17]. The catalyst particles were removed by washing with 32.5% nitric acid, which also leads to some oxidation of the p-MWCNT. Following purification p-MWCNT were filtered, washed with deionized water and dried at 110 °C for several hours [17]. They are composed of individual filaments of graphene walls with an average outer diameter and length of 11 and 980 nm, respectively [15]. To generate MWCNT-OH, hydroxyl groups were attached to the surface by 1,3-dipolar cycloaddition with tricine and DHBA as reactants (Fig. 1) [11]. For synthesis of MWCNT-COOH (Fig. 1), 200 mg p-MWCNT were dispersed in H_2_SO_4_/HNO_3_ (3:1) for 3 h in a sonication bath, filtrated and washed with distilled H_2_O until the pH was neutral. The product MWCNT-COOH was dried overnight at 108 °C. Synthesis of MWCNT-NH_2_ and MWCNT-SH was performed as described by Jeong et al. with an extended reaction time (Fig. 1) [18]. Briefly, 10 mg MWCNT-COOH and 10 mg DCC were sonicated for 5 min in 2 ml THF and stirred for 30 min. The dispersion was filtrated and washed with THF and methanol. Thereafter, the activated MWCNT were dispersed in THF. To produce MWCNT-NH_2_ and MWCNT-SH 4 ml ethylenediamine in 2 ml THF and 25 mg cysteamine in 4 ml THF, respectively, were added and the mixtures were stirred for 2.5 h. Afterwards, the dispersions were filtrated and washed with THF. The final products were dried for 72 h at 40 °C.

Finally, pristine and functionalized MWCNT were analyzed by X-ray photoelectron spectroscopy (XPS), thermogravimetric analysis (TGA), Fourier transform infrared spectroscopy (FT-IR) and Raman spectroscopy as described previously [11, 15]. XPS experiments were carried out in an ultrahigh vacuum system equipped with the hemispherical electron analyzer PHOIBOS 100 (SPECS, Berlin, Germany) operating at constant pass energy (15 eV). The photoelectrons were excited with non-monochromatic Mg Kα (1253.6 eV) radiation. The X-ray source was run at a power of 300 W. The powder materials were fixed in special sample holders with molds of 4 mm diameter. The analysis region was approximately 1 mm in diameter and the resolution was 0.2 eV. TGA was carried out in a SDT Q600 (TA Instruments, Eschborn, Germany) with a heating rate of 10 K/min and a synthetic air flow of 100 ml/min. FT-IR spectra were recorded at room temperature in transmission mode with an IFS 113v spectrometer (Bruker, Karlsruhe, Germany) with a resolution of 0.5 cm^−1^. The Raman spectroscopy measurements were performed using a Raman–Fourier-transform-spectrometer DXR SmartRaman (Thermo Fisher Scientific) at a wavelength of 532 nm and a laser power of 8 mW. The resolution was 1 cm^−1^.

Stock dispersions of the MWCNT (1 mg/ml) in phosphate buffered saline (PBS; pH 7.4) were prepared by sonication for 30 min in an ultrasonic bath. To compare the dispersion stability of the pristine and functionalized MWCNT, dispersions with a MWCNT concentration of 1 mg/ml were stored at room temperature for 24 h or three months. MWCNT dilutions for the cellular assays were prepared from the stock dispersions using cell culture medium.

### Cell culture, MWCNT treatment and cell viability assay

The human BCa cell line EJ28 (University of Frankfurt, Frankfurt, Germany) was cultured in Dulbecco’s modified eagle’s medium (DMEM; 4.5 g/l glucose) with 10% fetal calf serum, 1% gentamicin and 1% non-essential amino acids (all from GE Healthcare, Munich, Germany) in a humidified atmosphere containing 5% CO_2_ at 37 °C. After seeding 300 cells per well in 96well plates and adherence for 72 h, cells were treated with 0.1 mg/ml MWCNT dispersions in DMEM. 24 h after treatment the medium was changed. Cell viability was examined in quadruplicates 48, 72 and 96 h after MWCNT treatment start using the cell proliferation reagent WST-1 (Roche, Mannheim, Germany) according to the manufacturer’s protocol.

### Transmission electron microscopy of MWCNT uptake into cells

The cellular uptake of MWCNT was investigated by transmission electron microscopy (TEM) studies. 100,000 EJ28 cells were seeded in 25 cm^2^-flasks. After 72 h adherent cells were treated with 0.1 mg/ml MWCNT. Cells without MWCNT treatment served as control. After 24 h incubation cells were harvested by trypsin/EDTA treatment. TEM samples were prepared by the Institute of Pathology (TU Dresden, Dresden, Germany) as described previously and examined with an EM900 transmission electron microscope (Zeiss, Jena, Germany) [11]. Then, the localization of the MWCNT inside the cells was determined and the amount of cells showing a cellular uptake was estimated.

### Mucoadhesion studies

Mucoadhesive properties of the different functionalized MWCNT were evaluated as described previously [15]. Briefly, explanted mouse bladders (animal facility, Medical Faculty, TU Dresden, Dresden, Germany) were incubated with the various MWCNT types (0.2 mg/ml) for 1 h at room temperature using Franz diffusion cells (GauerGlas, Püttlingen, Germany). Subsequently, tissue sections were prepared from formalin-fixed paraffin-embedded tissues, stained with hematoxylin–eosin (HE) and evaluated by light microscopy. Percentage of urothelial area covered by MWCNT was considered in relation to total urothelial area by using the software ImageJ (Version 1.4.3.67, Broken Symmetry Software).

### Binding of CS-ODN to MWCNT and AS-ODN hybridization

CS-ODN, AS-ODN and non-complementary nonsense ODN (NS-ODN) as well as amino-group modified CS-ODN (NH_2_-CS-ODN) and maleimide-functionalized CS-ODN (Mal-CS-ODN) were purchased from biomers.net (Ulm, Germany); the respective sequences are listed in Table 1. The selected AS-ODN were targeted at the angiogenic growth factor VEGF [19] and CS-ODN represent the appropriate complementary sequence to the AS-ODN. The sequence of the NS-ODN was designed in that way that there is no binding to the CS-ODN, which was verified by using the software mfold 2.3 (http://unafold.rna.albany.edu/?q=mfold).

**Table 1:** Sequences and intended use of synthetic oligodeoxynucleotides

Type	Sequence	Intended use and remarks
CS-ODN	5′-ACG CTG CCG CCA CCA CAC CA-3′	Adsorbed to pristine and modified MWCNT
NH_2_-CS-ODN	5′-NH_2_-C_6_-ACG CTG CCG CCA CCA CAC CA-3′	Covalently bound to EDC/NHS-activated MWCNT-COOH; following activation with DSS covalently bound to MWCNT-NH_2_
Mal-CS-ODN	5′-Mal-ACG CTG CCG CCA CCA CAC CA-3′	Covalently bound to MWCNT-SH
AS-ODN	5′-TGG TGT GGT GGC GGC AGC GT-3′	Therapeutic AS-ODN against the angiogenic growth factor VEGF, complementary to CS-ODN
NS-ODN	5′-CCA AAC CCG TCA ATC AAG TC-3′	Control, non-complementary to CS-ODN

*AS-ODN* antisense oligodeoxynucleotides, *CS-ODN* carrier strand oligodeoxynucleotides, *Mal* maleimide, *Mal-CS-ODN* maleimide-functionalized CS-ODN, *NH*_*2*_ *-CS-ODN* amino-group modified CS-ODN, *NS-ODN* nonsense oligodeoxynucleotides

In order to covalently bind CS-ODN to the modified MWCNT, specific activation procedures which are frequently used for the respective modifications of the MWCNT had to be conducted (Table 1). In the following procedures, the concentration of sodium chloride and the ionic strength of the buffer solutions were kept constant in order to have the same ionic conditions in all buffer systems. Furthermore, 1 mg MWCNT was firstly dispersed in 1 ml PBS (pH 7.4) for 30 min in an ultrasonic bath and then the respective amount of MWCNT was used for the binding of CS-ODN.

#### Adsorption of CS-ODN to pristine and modified MWCNT

0.5 nmol CS-ODN were mixed with 50 µg MWCNT in 0.5 ml PBS and then shaken at room temperature overnight. Afterwards, the excess of CS-ODN was removed by centrifugation over a centrifugal filter (with modified nylon, pore size 0.2 µm; VWR International GmbH) for 1 min at 2300 rcf followed by two washing steps with 0.5 ml PBS.

#### EDC/NHS-activation of MWCNT-COOH and coupling with NH_2_-CS-ODN

50 µg MWCNT-COOH were activated with 100 mM EDC and 100 mM NHS in 0.5 ml MES (pH 6.0, 100 mM) by shaking the mixture for 1 h at room temperature. The buffer was removed by separation of the MWCNT-COOH via centrifugation over a centrifugal filter for 1 min at 2300 rcf. Afterwards, the activated MWCNT-COOH were mixed with 0.5 nmol NH_2_-CS-ODN in 0.5 ml potassium phosphate buffer (pH 8.0) and shaken at room temperature overnight followed by the removal of the excess CS-ODN.

#### Coupling of DSS-activated NH_2_-CS-ODN to MWCNT-NH_2_

25 µl of a 1 mM NH_2_-CS-ODN solution in water was mixed with 7.5 µl NaHCO_3_-buffer (pH 8.6, 1 M) and 25 µl DSS (10 mg/ml in DMSO). Excess DSS was separated using an illustra™ NAP™-10 sephadex™ G-25 column (GE Healthcare, Munich, Germany) and activated ODN were concentrated by Amicon^®^ Ultra centrifugal filters (3000 MWCO; Merck, Darmstadt, Germany). 0.5 nmol of the activated NH_2_-CS-ODN were mixed with 50 µg MWCNT-NH_2_ in 0.5 ml PBS and shaken at room temperature overnight followed by the removal of the excess CS-ODN.

#### Coupling of Mal-CS-ODN to MWCNT-SH

0.5 nmol Mal-CS-ODN were mixed with 50 µg MWCNT-SH in 0.5 ml PBS and shaken at room temperature overnight followed by the removal of the excess CS-ODN.

#### AS-ODN hybridization

AS-ODN or NS-ODN (0.1 nmol in 200 µl PBS) were added onto the centrifugal filters containing 50 µg MWCNT and allowed to hybridize for 30 min. The excess of AS-ODN or NS-ODN from the ODN-MWCNT-mixtures was removed by centrifugation over a centrifugal filter for 1 min at 2300 rcf followed by two washing steps with 0.5 ml PBS.

The aforementioned procedures resulted in the following maximum concentrations of ODN per mg CNT: 10 nmol/mg_CNT_ for CS-ODN and 2 nmol/mg_CNT_ for AS-ODN and NS-ODN.

### Fluorescence measurements for binding and release studies

For binding and release studies with the subsequent fluorescence measurements the respective CS-ODN were labeled at the 3′-end, whereas AS-ODN and NS-ODN were labeled at the 5′-end with 6-carboxyfluorescein (6-FAM) for release in PBS or Dy-557 for release in NaCl and urine. Following binding of the CS-ODN to the various MWCNT the supernatant and the two washing solutions were used for fluorescent measurements. For the release experiments at experimental conditions, 0.5 ml PBS was given to the various MWCNT with attached ODN on the centrifugal filter. The release was carried out at 80 °C for 10 min (1. release) and for another 10 min (2. release). The release at physiological conditions was carried out in 0.9% NaCl solution and pure urine as well as mixtures thereof at 37 °C for 1 h (1. release) and for 24 h (2. release). Afterwards, the solutions were centrifuged through the centrifugal filter again to remove the released ODN. The fluorescent measurements of the supernatant, the washing solutions and the release solutions were performed in a precision cuvette made from quartz glass Suprasil^®^ (Hellma GmbH & Co. KG, Müllheim, Germany) with the F-4500 FL spectrophotometer from Hitachi (Tokio, Japan) with time scan mode for 60 s. The excitation and emission wavelengths for 6-FAM were λ = 494 and λ = 515 nm, respectively. For Dy-557 the excitation wavelength was λ = 555 nm and the emission wavelength was λ = 576 nm.

### Statistical analyses

All experiments were reproduced independently at least twice. Data are presented as mean ± standard deviation, if not otherwise indicated. Outliers were identified by using the Dixon test and were not considered for averaging.

## Results

### Characterization of pristine and functionalized MWCNT

XPS spectra of all samples were measured and the elemental distribution was calculated (Table 2). p-MWCNT did not contain any nitrogen and sulfur. Due to the washing procedure of the sample with nitric acid to remove catalyst particles and amorphous carbon, the p-MWCNT were slightly oxidized resulting in an oxygen content of 1.8%. Compared to p-MWCNT, MWCNT-OH showed an increased content of oxygen (7.3%) and nitrogen (3.2%) based on the additional 1,3-dipolar cycloaddition. The sonication in nitric and sulfuric acid increased the oxygen content up to 12.4% for MWCNT-COOH and incorporated also some nitrogen into the sample (0.4%). This acid treatment formed different oxygen-containing functional groups on the surface of the MWCNT-COOH such as hydroxyl and aldehyde groups, but mainly carboxyl groups. The amidation of MWCNT-COOH with ethylenediamine resulted in MWCNT-NH_2_ with an increased nitrogen content (4.1%) combined with a decreased oxygen content (8.5%), because OH-groups were exchanged for NH-CH_2_-CH_2_-NH_2_ substituents. MWCNT-SH behaved similar to MWCNT-NH_2_. The sulfur content of 1.3% indicated a successful reaction.

**Table 2:** Results of XPS measurements of pristine and functionalized MWCNT

Sample	C 1s [%]	N 1s [%]	O 1s [%]	S 2s [%]	Functionalization degree [nmol/mg_CNT_]
p-MWCNT	98.2	0.0	1.8	0.0	–
MWCNT-OH	89.5	3.2	7.3	0.0	4.4
MWCNT-COOH	86.1	0.4	12.4	0.0	4.9
MWCNT-NH_2_	87.4	4.1	8.5	0.0	1.5
MWCNT-SH	89.4	2.3	7.0	1.3	1.0

The highest overall functionalization degrees were detected for MWCNT-OH (4.4 nmol/mg_CNT_) and MWCNT-COOH (4.9 nmol/mg_CNT_) (Table 2). In MWCNT-OH five hydroxyl groups were added simultaneously and thus contributed to the high functionalization degree. The functionalization values for MWCNT-NH_2_ (1.5 nmol/mg_CNT_) and MWCNT-SH (1.0 nmol/mg_CNT_) were lower indicating that not all carboxyl groups had been substituted. Furthermore, cysteamine had only one amino group to react with the activated carboxyl group, whereas ethylenediamine had two. This could contribute to the even lower functionalization degree of MWCNT-SH compared to MWCNT-NH_2_.

TGA measurements (Additional file 1: Figure S1A) showed that p-MWCNT were stable until a temperature of 550 °C and then started to decompose completely to CO_2_ within a temperature range of 550–650 °C. In contrast, the thermal stability of MWCNT-COOH, MWCNT-NH_2_ and MWCNT-SH was given until a temperature of 150 °C and between 150 and 250 °C they started to oxidize slowly. MWCNT-OH were stable until 250 °C and then slowly oxidized in a temperature range of 250–320 °C. This much lower thermal stability of all functionalized MWCNT can be attributed to the more unstable functional groups on their surface. Pristine and functionalized MWCNT started to decompose quickly at 550–650 °C. For MWCNT-NH_2_ and MWCNT-SH the weight loss before degradation of the MWCNT backbone was higher than for MWCNT-COOH due to the attached groups on the surface of MWCNT-NH_2_ and MWCNT-SH, which oxidize at lower temperatures.

The FT-IR spectra (Additional file 1: Figure S1B) of all MWCNT showed bands at 3435, 1634 and 1384 cm^−1^ corresponding to O–H, C=O and C–O bonds, respectively. In addition, MWCNT-NH_2_ and MWCNT-SH exhibited another C=O stretching vibration (amide group) at 1580 cm^−1^, which confirmed the successful amidation with ethylenediamine or cysteamine of these samples.

The Raman spectra (Additional file 1: Figure S1C) showed for all samples the two standard peaks at 1350 cm^−1^ (G-band) and 1591 cm^−1^ (D-band), which corresponded to graphitic and non-graphitic carbon, respectively. The ratio between the G-band and the D-band did not change during the functionalization processes. Raman spectroscopy was not sensitive enough to visualize the differences in the samples.

In order to examine the dispersibility of the MWCNT and the long-term behavior of their dispersions, MWCNT in PBS were sonicated followed by a monitoring of the dispersions. Homogenous dispersions were only obtained for the functionalized MWCNT (Additional file 1: Figure S2). In contrast, p-MWCNT could not be dispersed sufficiently in PBS and consequently coarse agglomerates were observed. 24 h after sonication, sedimentation of all dispersions was detected with p-MWCNT showing the highest sedimentation rate. The functionalized MWCNT could easily be converted back into dispersion by soft agitation of the sample vessel. After incubation for three months, all samples showed the same sedimentation behavior as observed at the 24 h time point.

### Impact of pristine and functionalized MWCNT on the cell viability of EJ28 BCa cells

**Fig. 2:**
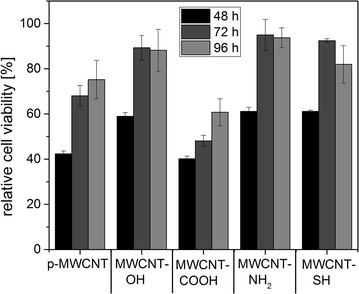
Time-dependent influence of pristine and functionalized MWCNT on the relative cell viability of EJ28 cells. Cells were treated for 24 h with 0.1 mg/ml MWCNT and then cellular viability was measured 48, 72 and 96 h after treatment start by using the WST-1 assay. Untreated cells served as control (100%)

Next, the impact of 0.1 mg/ml pristine and functionalized MWCNT on cellular viability was tested in EJ28 BCa cells. 48 h after treatment start, all MWCNT modifications caused a considerable inhibition of cellular viability (Fig. 2), whereupon MWCNT-COOH and p-MWCNT caused the strongest reduction of the relative cell viability down to about 40%. In contrast, the relative cell viability following treatment with MWCNT-OH, MWCNT-NH_2_ and MWCNT-SH was approximately 60% after 48 h. However, the relative cell viability increased over the investigated time period resulting in the lowest inhibition for all MWCNT modifications 96 h after treatment start. At this time point, the relative cell viability was 60% for MWCNT-COOH, 75% for p-MWCNT and between 82 and 94% for MWCNT-OH, MWCNT-NH_2_ and MWCNT-SH. The detrimental impact of the various MWCNT on the cell viability was as follows: MWCNT-COOH > p-MWCNT > MWCTNs-OH/MWCNT-NH_2_/MWCNT-SH.

### Cellular uptake of pristine and functionalized MWCNT

**Fig. 3:**
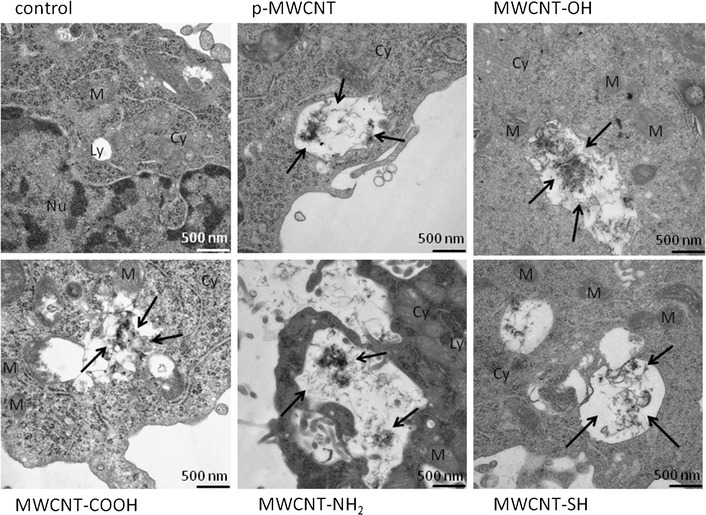
Internalization of pristine and functionalized MWCNT by EJ28 cells as visualized by TEM. Representative TEM images of EJ28 cells after incubation with 0.1 mg/ml MWCNT for 24 h are depicted. Cells without MWCNT treatment served as control. *Black arrows* indicate MWCNT inside the cells. Cy: cytoplasm; Ly: lysosome; M: mitochondrion; Nu: nucleus

Additionally, the cellular uptake of MWCNT was investigated with TEM analysis (Fig. 3). The vacuoles of untreated cells were significantly smaller than the vacuoles of treated cells. The MWCNT taken up by the cells could be mostly found as agglomerates in the vacuoles. No MWCNT were located in the cell nuclei. After treatment with p-MWCNT 5–10% of cells showed an uptake, whereas for MWCNT-COOH and MWCNT-NH_2_ 30–35% of cells exhibited a MWCNT uptake. MWCNT-SH showed the lowest uptake with 5% of cells having MWCNT-SH inside their vacuoles. After treatment with MWCNT-OH, 40% of cells showed an uptake, whereupon 90% of the incorporated MWCNT-OH could be found in vacuoles and 10% were included directly in the cytoplasm of cells. MWCNT-OH in the cytoplasm were individualized and not agglomerated like in the vacuoles (Additional file 1: Figure S3).

### Mucoadhesive potential of pristine and functionalized MWCNT

**Fig. 4:**
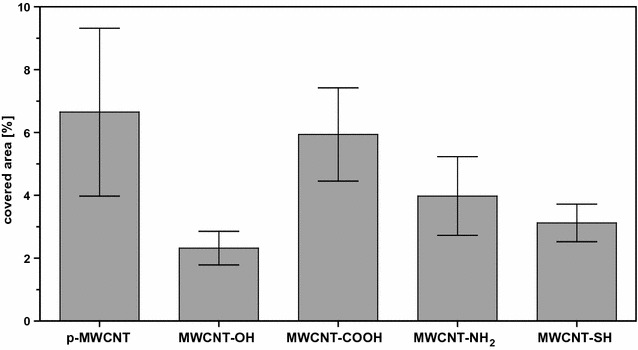
Mean percentage of urothelial area covered by pristine or functionalized MWCNT. Explanted mouse bladders were incubated with the various MWCNT types (0.2 mg/ml) for 1 h at room temperature using Franz diffusion cells. Subsequently, the coverage of urothelium by MWCNT was evaluated on HE-stained tissue sections by light microscopy. Results are depicted as mean ± standard error

Next, ex vivo mucoadhesion experiments of the pristine and modified MWCNT were performed on explanted mouse bladders. All MWCNT types displayed only an adhesion to the urothelium and did not penetrate into deeper tissue layers. Furthermore, the surface of the mouse bladders showed no visible signs of damage following incubation with MWCNT. The mean percentages of MWCNT-covered surface areas along the urothelium were estimated to evaluate the mucoadhesive properties of the different modified MWCNT types (Fig. 4) and ranged from 2.3% for MWCNT-OH to 5.9% for MWCNT-COOH. The unmodified p-MWCNT showed a slightly higher mean percentage (6.7%) of covered surface area of the urothelium than the modified MWCNT.

### Binding of CS-ODN to MWCNT

In the present study, two different strategies were employed in order to attach CS-ODN to the MWCNT: via non-covalent adsorption and covalent binding. Because a fluorescence quenching occurred when measuring fluorescent-labeled CS-ODN directly on the surface of MWCNT, the amount of CS-ODN coupled to MWCNT could only be measured indirectly. By measuring the amount of CS-ODN in the supernatant of the binding reaction and in the washing solutions an estimation of coupled CS-ODN to the surface of MWCNT was possible. A high concentration of CS-ODN in these solutions would indicate a poor attachment to the MWCNT. Furthermore, a release study was performed at 80 °C, because it has been shown that non-covalent interactions are not stable at higher temperatures resulting in a release of the CS-ODN [20].

**Fig. 5:**
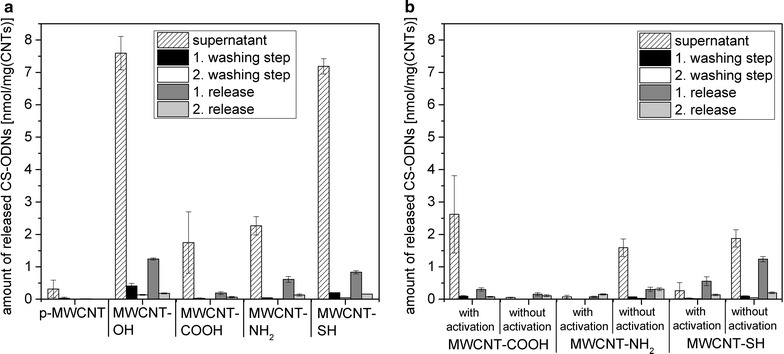
Release of CS-ODN attached to pristine or functionalized MWCNT. Depicted is the amount of released CS-ODN following **a** adsorption or **b** covalent binding with and without prior activation to pristine or modified MWCNT. The initial concentration of the 6-FAM-labeled CS-ODN was 10 nmol/mg_CNT_ and the released amount was determined by fluorescence measurements in the supernatant of the binding reaction, in the washing solution (twice with PBS) and in the release solution (in PBS at 80 °C for 2 × 10 min). The specific activation procedures prior to the covalent binding were as follows: NH_2_-CS-ODN coupled to MWCNT-COOH activated with EDC/NHS, DSS-activated NH_2_-CS-ODN coupled to MWCNT-NH_2_, maleimide-activated CS-ODN coupled to MWCNT-SH. Control experiments were performed without specific activation

First, the non-covalent adsorption of CS-ODN to pristine and modified MWCNT was investigated (Fig. 5a). The lowest amount of CS-ODN in the supernatant was achieved for p-MWCNT with 0.30 nmol/mg_CNT_. Additionally, there were no CS-ODN in the washing and releasing solutions. Altogether, this indicates a good adsorption capacity and stability for CS-ODN to p-MWCNT. The highest amount of CS-ODN in the supernatant was measured for MWCNT-OH with 7.60 nmol/mg_CNT_ followed by 7.20 nmol/mg_CNT_ in the supernatant after CS-ODN-adsorption to MWCNT-SH. Additionally, there was a rather high release at 80 °C for these MWCNT modifications. Therefore, the major part of the initially added CS-ODN (10 nmol/mg_CNT_) was not coupled to the surface of MWCNT-OH and MWCNT-SH. For MWCNT-NH_2_ and MWCNT-COOH the CS-ODN concentration in the supernatant was 2.30 and 1.70 nmol/mg_CNT_, respectively. The amount of CS-ODN in the washing and release solutions was also lower compared to MWCNT-OH and MWCNT-SH. The adsorption capacity and stability of CS-ODN to MWCNT reflected by a decreasing amount CS-ODN in the supernatant, washing and releasing solutions were as follows: p-MWCNT > MWCNT-COOH > MWCNT-NH_2_ > MWCNT-SH > MWCNT-OH.

Next, functionalized MWCNT were used for the covalent coupling of fluorescent-labeled CS-ODN. First, the carboxyl-groups of MWCNT-COOH were activated by EDC/NHS-treatment, so that they then could react with the amino-groups of amino-group-modified CS-ODN (NH_2_-CS-ODN). The amount of NH_2_-CS-ODN in the supernatant after EDC/NHS-activation and in the first release solution was about 2.60 and 0.30 nmol/mg_CNT_, respectively, indicating a moderate to good binding capacity and stability (Fig. 5b). Surprisingly, the amount of NH_2_-CS-ODN in the supernatant and after the release was nearly zero for NH_2_-CS-ODN coupled to MWCNT-COOH without EDC/NHS-activation reflecting a better binding capacity and stability than with EDC/NHS-activation.

NH_2_-CS-ODN were activated by DSS and used for a covalent binding to the amino-groups of MWCNT-NH_2_. In comparison to the non-covalent binding of NH_2_-CS-ODN without DSS-activation to MWCNT-NH_2_, the amount of NH_2_-CS-ODN in the supernatant and after release for DSS-activated NH_2_-CS-ODN to MWCNT-NH_2_ was much lower (Fig. 5b): 0.07 vs 1.60 nmol/mg_CNT_ and 0.07 vs 0.30 nmol/mg_CNT_, respectively. This indicates a specific binding reaction and strength for the DSS-activated NH_2_-CS-ODN to MWCNT-NH_2_.

For maleimide-functionalized CS-ODN (Mal-CS-ODN), which react with thiol groups of MWCNT-SH, the situation was similar (Fig. 5b). The direct comparison of Mal-CS-ODN coupled to MWCNT-SH and CS-ODN without maleimide-functionalization adsorbed to MWCNT-SH showed a CS-ODN concentration of 0.30 and 1.90 nmol/mg_CNT_, respectively, in the supernatants. Also, the CS-ODN amount in the releasing solutions was lower for Mal-CS-ODN coupled to MWCNT-SH compared to CS-ODN without maleimide-functionalization. This demonstrates that Mal-CS-ODN could be covalently and specifically coupled to MWCNT-SH resulting in a good binding stability.

The binding capacity and stability of CS-ODN to the modified MWCNT after specific activation were as follows: MWCNT-NH_2_ > MWCNT-SH > MWCNT-COOH.

### Hybridization of AS-ODN to CS-ODN-MWCNT and release at experimental conditions

**Fig. 6:**
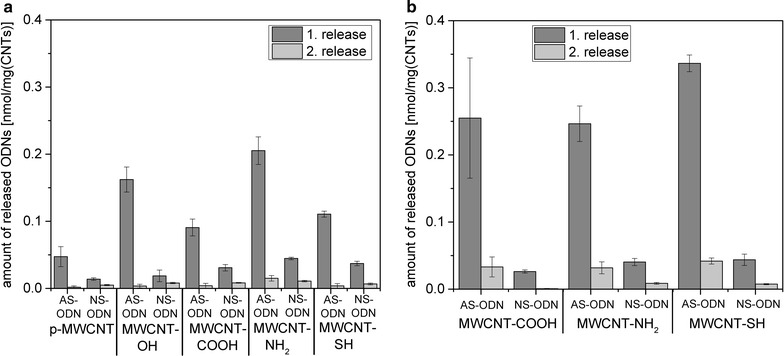
Release of AS-ODN or NS-ODN following hybridization to CS-ODN attached to pristine or functionalized MWCNT. Depicted is the amount of released AS-ODN or NS-ODN following hybridization to **a** adsorbed or **b** covalently bound CS-ODN to pristine or modified MWCNT. The initial concentration of the 6-FAM-labeled AS-ODN and NS-ODN was 2 nmol/mg_CNT_ and the released amount was determined by fluorescence measurements in the release solution (in PBS at 80 °C for 2 × 10 min). The specific activation procedures prior to the covalent binding were performed analog to Fig. 2. Non-complementary NS-ODN served as control

After adsorption or covalent coupling of CS-ODN to MWCNT, the complementary AS-ODN could hybridize to them. Non-complementary NS-ODN served as control to show the specificity of the AS-ODN-hybridization to the CS-ODN. Afterwards, the release of AS-ODN and NS-ODN was performed at 80 °C, which is above the melting temperature of AS-ODN and NS-ODN ensuring a high release rate. Regardless of the attachment method of the CS-ODN to the MWCNT, most of the AS-ODN were already released after 10 min at 80 °C in PBS (1. release) and only small amounts of AS-ODN were released after additional 10 min (2. release; Fig. 6). The first release of AS-ODN from CS-ODN which were adsorbed to MWCNT was highest for MWCNT-NH_2_ (0.20 nmol/mg_CNT_) followed by MWCNT-OH (0.16 nmol/mg_CNT_), MWCNT-SH (0.11 nmol/mg_CNT_) and MWCNT-COOH (0.09 nmol/mg_CNT_) (Fig. 6a). The lowest release of AS-ODN with 0.05 nmol/mg_CNT_ was achieved for p-MWCNT (Fig. 6a), although they had the highest amount of adsorbed CS-ODN as aforementioned. The release of AS-ODN from CS-ODN covalently bound to MWCNT-COOH (0.26 nmol/mg_CNT_), MWCNT-NH_2_ (0.25 nmol/mg_CNT_) or MWCNT-SH (0.34 nmol/mg_CNT_) were similar amongst these MWCNT with MWCNT-SH showing a slightly higher release rate (Fig. 6b). For all samples the release of NS-ODN was clearly lower than for AS-ODN indicating the specific hybridization of AS-ODN to the CS-ODN at the MWCNT surface (Fig. 6).

While comparing the release of NS-ODN, which can only adsorb to the MWCNT, with the amount of released AS-ODN it is possible to estimate the ratio of unspecific adsorption for AS-ODN. The difference between the release of NS-ODN and AS-ODN, which corresponds to the hybridization rate of AS-ODN, was highest for MWCNT-OH. In detail, eight times more AS-ODN than NS-ODN were released from MWCNT-OH, which corresponds to a adsorption rate of AS-ODN to MWCNT-OH of 12.5% compared to NS-ODN with an estimated adsorption rate of 100%. The estimated adsorption rate of AS-ODN was 20% for MWCNT-NH_2_ and 33% for p-MWCNT, MWCNT-COOH and MWCNT-SH. Consequently, CS-ODN adsorbed to MWCNT-OH and MWCNT-NH_2_ had the highest hybridization rate of AS-ODN.

### Release of AS-ODN at physiological conditions in urine

**Fig. 7:**
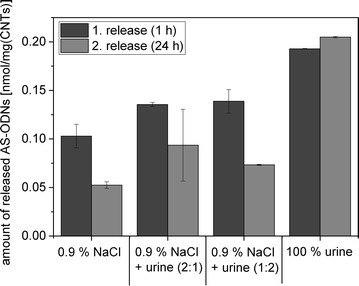
Release of AS-ODN following hybridization to CS-ODN adsorbed to MWCNT-OH at physiological conditions. The initial concentration of the Dy-557-labeled AS-ODN was 2 nmol/mg_CNT_ and the released amount was determined by fluorescence measurements in the release solutions. The release studies were performed at 37 °C in 0.9% NaCl solution, urine and different dilutions thereof

For prospective medical applications MWCNT carrying therapeutic AS-ODN should be instilled into the urinary bladder in a physiological NaCl solution (0.9%). Therefore and exemplarily, the release of AS-ODN after hybridization to CS-ODN adsorbed to MWCNT-OH was investigated at 37 °C using 0.9% NaCl solution and pure urine as well as mixtures thereof (Fig. 7). The release of AS-ODN was lowest in the 0.9% NaCl solution and increased with an increasing portion of urine in the mixture. Accordingly, the release of AS-ODN was the highest in pure urine: 0.19 nmol/mg_CNT_ after 1 h and 0.20 nmol/mg_CNT_ after 24 h.

## Discussion

CNT may represent a viable option as gene therapy vectors, because they can be chemically tailored at the surface in order to improve drug binding, dispersibility, biocompatibility and/or targeting [6]. Furthermore, CNT are known to penetrate cell membranes and thus could unload their cargo intracellularly [6, 8]. Because p-MWCNT are usually difficult to disperse in aqueous solutions, various types of surface functionalization with hydrophilic moieties are employed not only to increase their dispersibility, but also to improve their biocompatibility. In the present study, p-MWCNT were functionalized with hydroxyl groups (MWCNT-OH) via 1,3-dipolar cycloaddition of tricine/DHBA and with carboxylic groups (MWCNT-COOH) by oxidation with nitric and sulfuric acid. The amino groups (MWCNT-NH_2_) were introduced by the formation of an amide bond between ethylene diamine and carboxylic groups of MWCNT-COOH, whereas cysteamine was used instead of ethylene diamine for the attachment of thiol groups (MWCNT-SH). Depending on the moiety the degree of functionalization ranged between 1.0 and 4.9 nmol/mg_CNT_. By increasing the functionalization degree the MWCNT showed a much lower thermal stability due to the more unstable functional groups on their surface. MWCNT-NH_2_ and MWCNT-SH started to decompose at lower temperatures in comparison to all other functionalized MWCNT, because the oxidation of these functional groups occurred at lower temperatures. Furthermore, the ratio between the G-band and the D-band did not change during the functionalization processes indicating that no further defects were introduced into the modified MWCNT.

The prerequisite for the biomedical application of MWCNT is the formation of stable dispersions without agglomerates or aggregates. Therefore, an initial assessment of the dispersibility and the long-term dispersion stability of the different MWCNT modifications was performed. The introduction of hydrophilic moieties to the MWCNT surface led to a superior dispersibility and enhanced dispersion stability compared to unmodified p-MWCNT. Notably, the functionalized MWCNT formed good dispersions in PBS without the help of known adjuvants such as serum albumin and polyethylene glycol [21]. In the present study, the MWCNT modifications MWCNT-OH, MWCNT-NH_2_ and MWCNT-SH exhibited a lower impact on the cellular viability of EJ28 BCa cells than oxidized MWCNT-COOH and unmodified p-MWCNT. Due to their hydrophobic surface, p-MWCNT are poorly dispersible in aqueous solutions and tend to agglomerate and thus induce higher cell-damaging effects than well-dispersed MWCNT as proven by various studies [22, 23]. In accordance with our results, various in vitro and in vivo studies revealed that acid-treated SWCNT and MWCNT with carboxyl groups caused a substantial degree of cytotoxicity and/or inflammatory response [22–25]. These strong toxic effects might be evoked by their high negative surface charge, which could lead to enhanced CNT–cell-interactions [24]. In contrast and similar to our findings, MWCNT-OH were demonstrated to be less toxic than p-MWCNT or MWCNT-COOH [23]. Furthermore, Muller et al. have shown that the presence of structural defects at the surface of MWCNT is associated with a diminished biocompatibility due to a higher reactivity of the defective MWCNT [26]. However, in the present study this should not be the case, because it has been shown by Raman spectroscopy that no further defects have been introduced by the functionalization process. Moreover, it was shown that EJ28 BCa cells could recover over the investigated time period following incubation with pristine or modified MWCNT. In accordance, we could previously demonstrate that EJ28 BCa cells treated with different p-MWCNT varying in diameter and length could recover over time [15].

Furthermore, a general uptake of all investigated MWCNT into cells was observed. Cellular internalization into various cell types has already been shown for p-MWCNT, MWCNT-OH and MWCNT-COOH in our previous studies or by other groups [11, 16, 22, 23]. Here, the percentage of cells with internalized MWCNT ranged from 5 to 40% depending on the functional group of the MWCNT, whereupon MWCNT-OH had the highest and MWCNT-SH the lowest rates of internalization. The majority of MWCNT was located as agglomerates inside vacuoles, which were hence enlarged due to the uptake of MWCNT. This indicates that MWCNT were incorporated into the cells as cellular vesicles by energy-dependent endocytosis, which is one possibility for the uptake of CNT [8, 27]. Another possibility is the passive uptake by nanopenetration leading to the location of the MWCNT directly in the cytoplasm of cells [8, 27]. Of all investigated MWCNT within the present work, this could only be shown for MWCNT-OH. Ursini et al. also demonstrated that MWCNT-OH were localized freely in the cytoplasm and inside vacuoles, whereas MWCNT-COOH were only found inside vacuoles [23]. Furthermore, pristine and modified MWCNT were able to adhere to the urothelium of explanted mouse bladders. Similar to our previous findings with various p-MWCNT [15], all tested MWCNT types did not penetrate into deeper tissue layers and induced no visible signs of damage on the surface of the mouse bladders. However, the amount of the covered area was rather low (2–7%) and thus it was not possible to clearly identify the superiority of one modification over the other regarding their mucoadhesive potential. In a previous study by our group, different types of p-MWCNT led to similar percentages of covered urothelium from mouse bladders (5–10%) [15]. Apart from the present and our previous study, the mucoadhesive potential with regard to bladder treatment has so far only been investigated for nanoparticles other than CNT using in vitro or ex vivo methods [28–35]. CNT could also be coated or functionalized with hydrophilic polymers such as gelatin or chitosan, which could further increase the dispersibility, biocompatibility and mucoadhesion [3, 36].

In addition to conventional drugs, MWCNT can also be loaded with therapeutic nucleic acids such as AS-ODN or siRNA [6–8]. AS-ODN, for instance, can be coupled to MWCNT via complementary carrier strands (CS-ODN), which have been either covalently or non-covalently bound to MWCNT beforehand. In the present study, we could successfully attach CS-ODN by adsorption and covalent binding to the various MWCNT types. In order to evaluate the binding capacity and stability of CS-ODN to MWCNT, the amount of unbound fluorescent-labeled CS-ODN was determined in the supernatant of the binding reaction, the washing and release solutions. This approach was necessary, because the fluorescence quenching effect of MWCNT hindered the direct quantification of bound CS-ODN. Adsorption of CS-ODN to p-MWCNT, MWCNT-COOH and MWCNT-NH_2_ as well as covalent coupling to MWCNT-NH_2_ and MWCNT-SH resulted in the best binding capacity and stability as indicated by low concentrations of CS-ODN in the supernatant, washing and releasing solutions.

However, it is difficult to distinguish between covalent and non-covalent binding of CS-ODN to the CNT. The results suggest that the binding of CS-ODN to MWCNT was easier achieved by adsorption, particularly for MWCNT-COOH. Covalent binding and adsorption are competing processes. Through its strong affinity and spontaneity the adsorption process is probably more dominant than the slower covalent reaction. This thesis was also confirmed, because p-MWCNT displayed the highest amount of bound CS-ODN. The presence of functional groups at the surface of MWCNT could possibly hinder the adsorption of CS-ODN to the functionalized MWCNT. Responsible forces for an enhanced non-covalent adsorption of CS-ODN to MWCNT may be London dispersion forces and π–π-interactions between aromatic nucleobases and the aromatic graphene rings of the MWCNT. Also, hydrogen bonds can be formed between nucleobases or the sugar-phosphate-backbone and hydroxyl-groups of MWCNT-COOH and MWCNT-OH or amino-groups of MWCNT-NH_2_. Because the aromatic rings of the nucleobases and the MWCNT surface are hydrophobic, there are hydrophobic interactions between both. Furthermore, electrostatic interactions could occur between negatively charged CS-ODN and the positively charged MWCNT-SH and MWCNT-NH_2_ at neutral pH. p-MWCNT and MWCNT-OH are uncharged at neutral pH, whereas MWCNT-COOH are negatively charged, which could cause a repulsion with CS-ODN.

Subsequently, therapeutic AS-ODN could be hybridized to and reversibly released from the CS-ODN coupled via both strategies to the functionalized MWCNT. At experimental conditions (80 °C, buffer), the release of AS-ODN from CS-ODN adsorbed to MWCNT was most effective from MWCNT-OH and MWCNT-NH_2_ followed by MWCNT-COOH and MWCNT-SH. The lowest release of AS-ODN was achieved for p-MWCNT despite the highest amount of adsorbed CS-ODN. The release of AS-ODN from CS-ODN covalently attached to MWCNT-COOH, MWCNT-NH_2_ or MWCNT-SH were similar and thus did not depend on the type of MWCNT functionalization. For all samples the specific hybridization of AS-ODN to the CS-ODN at the MWCNT surface was indicated by the much lower release rate of NS-ODN, which can only adsorb to the MWCNT. However, it was not possible to experimentally distinguish between hybridization of AS-ODN to CS-ODN and a potential adsorption of AS-ODN to the MWCNT surface. Therefore, it might be possible that AS-ODN also adsorbed to free positions at the MWCNT surface in addition to hybridization to CS-ODN. By comparing the release of NS-ODN, which can only adsorb to the MWCNT, with the amount of released AS-ODN it was possible to estimate the ratio of unspecific adsorption for AS-ODN. The highest hybridization rate of AS-ODN was observed for CS-ODN adsorbed to MWCNT-OH and MWCNT-NH_2_. This could in turn result in the superior release rate of AS-ODN from these MWCNT modifications, because hybridization is a reversible process and hybridized AS-ODN might be easier released than the additional adsorbed portion.

Moreover, we could exemplarily demonstrate that AS-ODN could be released following hybridization to CS-ODN adsorbed to MWCNT-OH at physiological settings (37 °C, urine). The release in urine and NaCl as well as mixtures thereof was carried out to investigate the clinical applicability of the AS-ODN release from MWCNT. 0.9% NaCl solution is normally used as clinical instillation solution. The release of AS-ODN was lowest in 0.9% NaCl, whereas with an increasing amount of urine the release of AS-ODN also increased. It is possible that the stability of the AS-ODN hybridization is higher in the NaCl solution, because there is a higher concentration of monovalent counterions, which shield the phosphate–phosphate repulsion inside the double helix. When the urea concentration increases, the electrostatic forces between the negatively charged DNA phosphate backbone and sodium ions are decreasing leading to an enhanced release of AS-ODN. Furthermore, there are hydrogen bonds preferentially formed between nucleobases and urea instead of complementary base pairing, which causes a destabilization of the double helix and a release of AS-ODN [37]. In summary, for a clinical application it will be advantageous, when there is no release during instillation with NaCl. This will ensure that the release of AS-ODN first starts inside the bladder, when an increasing amount of urea facilitates the destabilization of the AS-ODN/CS-ODN-bond.

To date, only a few groups have investigated CNT as delivery vectors for AS-ODN or ODN. Jia et al. could efficiently transport AS-ODN against telomerase into cell nuclei by means of polyethylenimine-functionalized MWCNT-COOH, which led to a much higher cellular apoptosis than cell transfection with naked AS-ODN [13]. AS-ODN against c-myc conjugated to MWCNT-NH_2_ modified with polyamidoamine dendrimers could mediate a substantial inhibition of cell growth and c-myc expression [14]. Furthermore, SWCNT-COOH served as efficient vector for the intracellular delivery of decoy ODN against NF-κB, which hence could significantly diminish the NF-κB-dependent gene expression [12]. In our previous study, AS-ODN against the angiogenic growth factor VEGF could be released after hybridization to CS-ODN adsorbed onto MWCNT-OH at physiological conditions (37 °C, buffer) at slightly basic pH values. MWCNT-OH could also mediate an uptake of AS-ODN into EJ28 BCa cells, which resulted in a marginal inhibition of cellular viability and VEGF expression [11]. However, other targets with more relevance to BCa such as survivin [38] and hTERT [39] could mediate more pronounced anti-proliferative effects upon inhibition through AS-ODN than VEGF. This should be investigated in future studies.

## Conclusions

We could successfully functionalize p-MWCNT with hydrophilic moieties (MWCNT-OH, -COOH, -NH_2_, -SH), which led to an improved dispersibility and dispersion stability as well as to an enhanced biocompatibility for most of the modifications. All MWCNT were internalized by BCa cells and could adhere to the urothelium of explanted mouse bladders, although the amount of the covered urothelial area was rather low. AS-ODN could be hybridized to CS-ODN which were either adsorbed or covalently bound to pristine or functionalized MWCNT. However, the reversible release of AS-ODN at experimental conditions (80 °C, buffer) was most effective from CS-ODN attached to functionalized MWCNT, whereas it was the least effective from CS-ODN attached to p-MWCNT. Furthermore, we could exemplarily demonstrate that AS-ODN hybridized to MWCNT-OH-bound CS-ODN could also be released at physiological settings (37 °C, urine). Taken together, MWCNT functionalized with hydrophilic moieties represent an interesting strategy for the intravesical treatment of BCa with therapeutic AS-ODN. Nevertheless, the mucoadhesive properties of MWCNT-based delivery systems and thus their dwell time at the urothelium have to be improved further possibly via coating or functionalization with hydrophilic polymers.

## Additional file

**Additional file 1: Figure S1.** (A) TGA curves, (B) FT-IR spectra and (C) Raman spectra of pristine and functionalized MWCNT. a.u.: arbitrary units; rel.: relative. **Figure S2.** Dispersibility and dispersion stability of pristine and functionalized MWCNT in PBS. Dispersions of MWCNT (1 mg/ml) were freshly prepared in PBS by sonication for 30 min. Images were taken immediately after sonication (0 h), after storing for 24 h at room temperature followed by soft agitation and after storing for another three months. **Figure S3.** Cytoplasmic localization of MWCNT-OH within EJ28 cells as visualized by TEM. Representative TEM images of EJ28 cells after incubation with 0.1 mg/ml MWCNT-OH for 24 h are depicted. The image in (B) represents a higher magnification of the area defined in (A). Black arrows indicate MWCNT inside the cells. Cy: cytoplasm; Ly: lysosome; M: mitochondrion; V: vacuole.

## Abbreviations

6-FAM: 6-carboxyfluorescein
AS-ODN: antisense oligodeoxynucleotides
a.u.: arbitrary units
BCa: bladder cancer
CNT: carbon nanotubes
CS-ODN: carrier strand oligodeoxynucleotides
Cy: cytoplasm
DCC: *N,N′*-dicyclohexylcarbodiimide
DHBA: 3,4-dihydroxybenzaldehyde
DMEM: Dulbecco’s modified eagle’s medium
DMF: *N*,*N*-dimethylformamide
DSS: disuccinimidyl suberate
EDC: 1-ethyl-3-(3-dimethylaminopropyl)carbodiimid
FT-IR: Fourier transform infrared spectroscopy
HE: hematoxylin–eosin
Ly: lysosome
M: mitochondrion
Mal: maleimide
MES: 2-(*N*-morpholino)ethanesulfonic acid
MWCNT: multi-walled carbon nanotubes
NHS: *N*-hydroxysuccinimid
NS-ODN: nonsense oligodeoxynucleotides
Nu: nucleus
ODN: oligodeoxynucleotides
PBS: phosphate buffered saline
p-MWCNT: pristine multi-walled carbon nanotubes
rel.: relative
RT: room temperature
siRNA: small interfering RNAs
SWCNT: single-walled carbon nanotubes
TEM: transmission electron microscopy
TGA: thermogravimetric analysis
THF: tetrahydrofuran
V: vacuole
XPS: X-ray photoelectron spectroscopy
