# Supplementary figures and images for: Systematic evaluation of oligodeoxynucleotide binding and hybridization to modified multi-walled carbon nanotubes
Source: J Nanobiotechnology. 2017 Jul 17;15:53. doi: 10.1186/s12951-017-0288-z (PMC5513106; doi:10.1186/s12951-017-0288-z)

**A**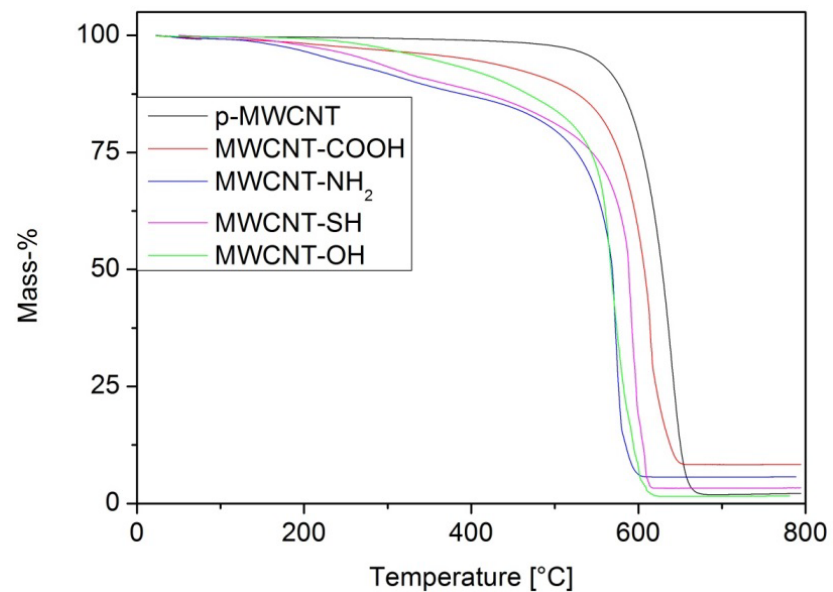**B**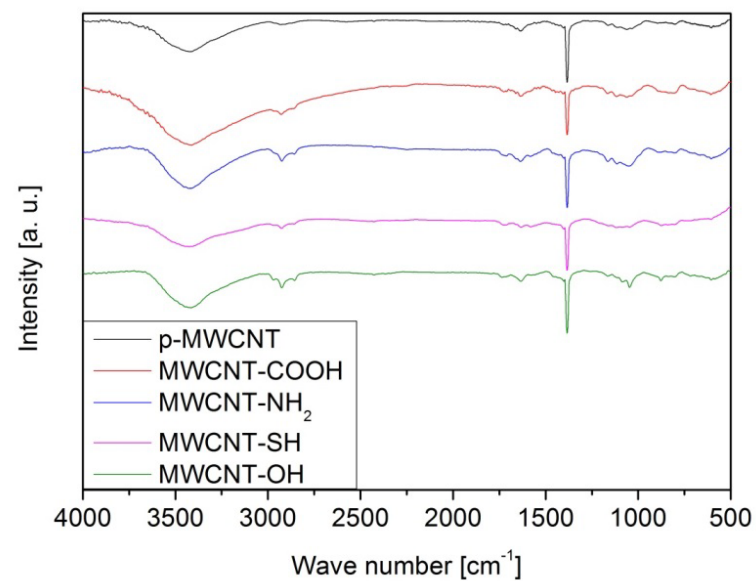**C**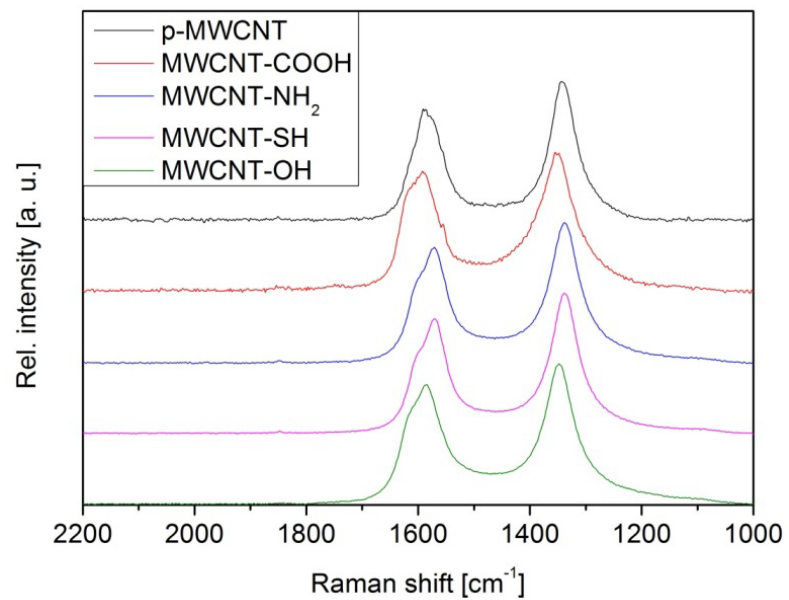

p-MWCNT

MWCNT-  
OH

MWCNT-  
COOH

MWCNT-  
NH<sub>2</sub>

MWCNT-  
SH

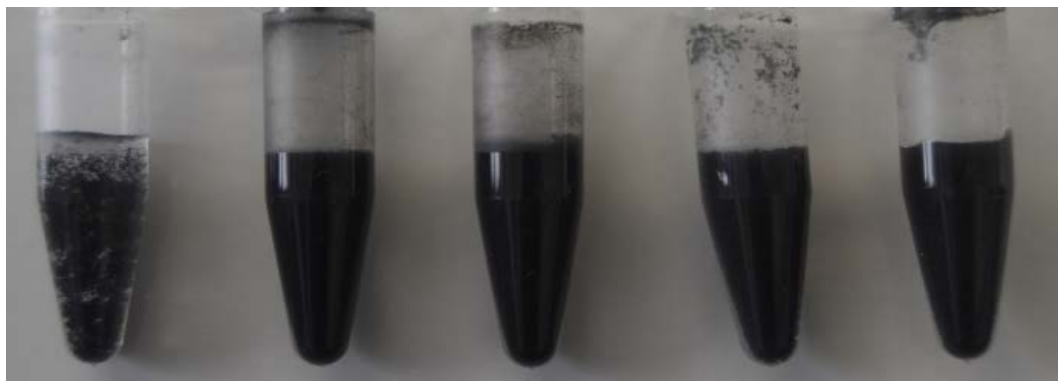

0 h

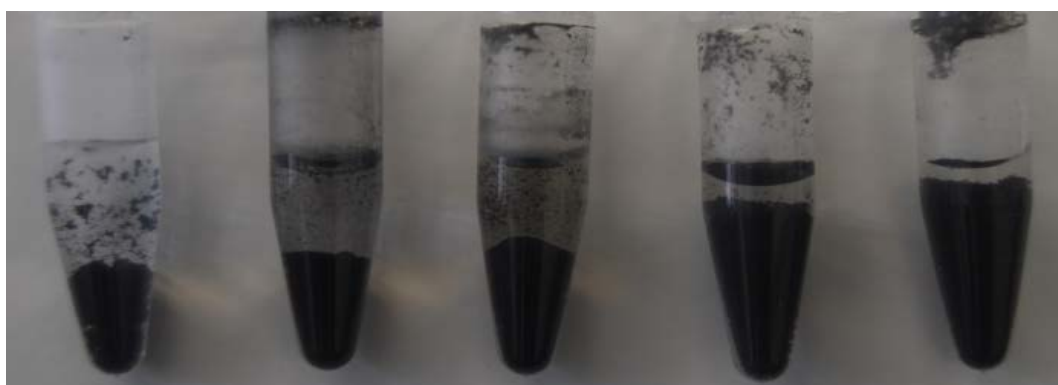

24 h

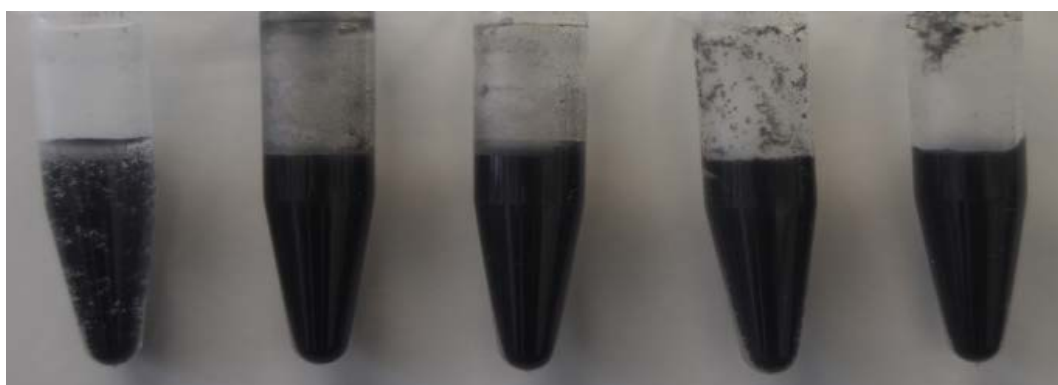

after soft  
agitation

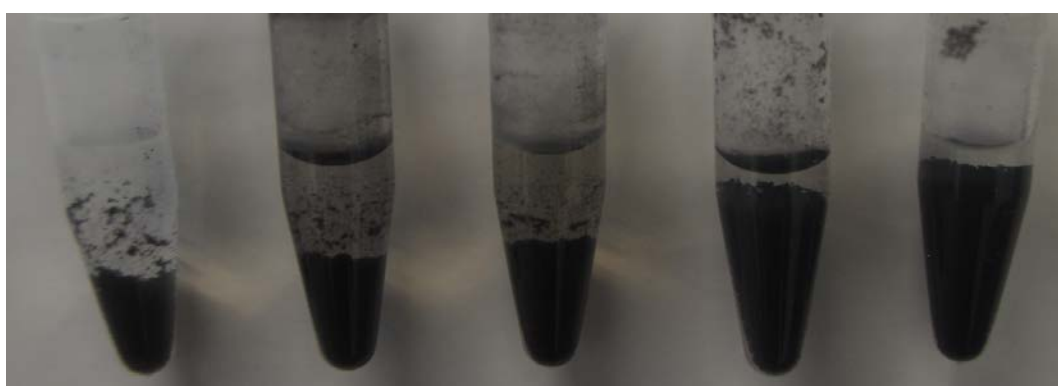

3 months

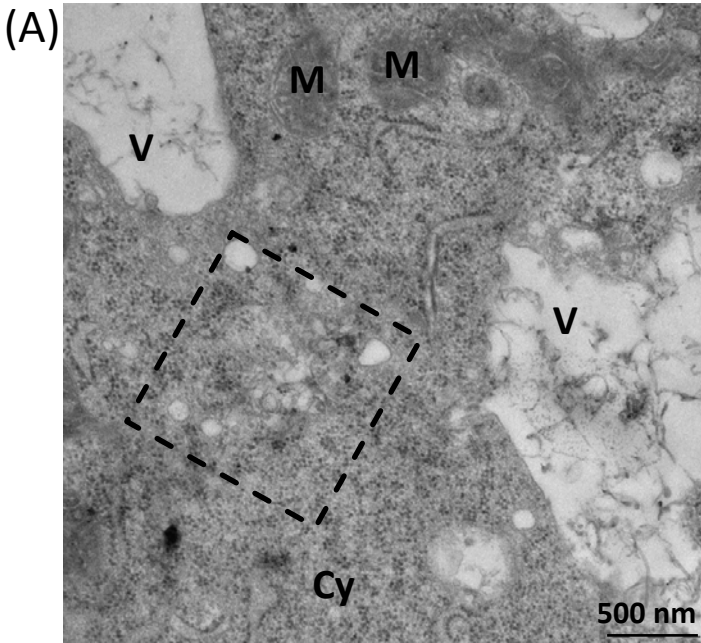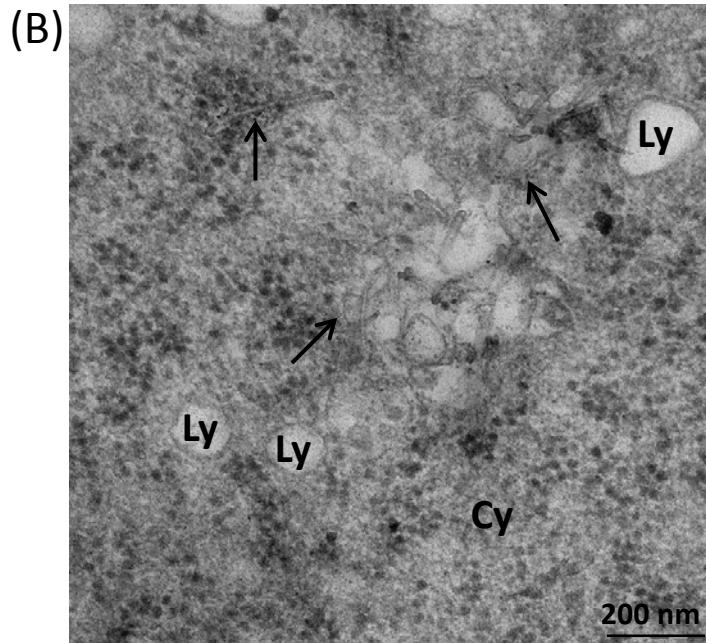

Supplement: Supplementary file 1 — Additional file 1: Figure S1. (A) TGA curves, (B) FT-IR spectra and (C) Raman spectra of pristine and functionalized MWCNT. a.u.: arbitrary units; rel.: relative. Figure S2. Dispersibility and dispersion stability of pristine and functionalized MWCNT in PBS. Dispersions of MWCNT (1 mg/ml) were freshly prepared in PBS by sonication for 30 min. Images were taken immediately after sonication (0 h), after storing for 24 h at room temperature followed by soft agitation and after storing for another three months. Figure S3. Cytoplasmic localization of MWCNT-OH within EJ28 cells as visualized by TEM. Representative TEM images of EJ28 cells after incubation with 0.1 mg/ml MWCNT-OH for 24 h are depicted. The image in (B) represents a higher magnification of the area defined in (A). Black arrows indicate MWCNT inside the cells. Cy: cytoplasm; Ly: lysosome; M: mitochondrion; V: vacuole. [file 12951_2017_288_MOESM1_ESM.pdf]
